# Atoms and the void: modular construction of ordered porous solids

**DOI:** 10.1038/s41467-020-18419-3

**Published:** 2020-09-16

**Authors:** James D. Wuest

**Affiliations:** Département de Chimie, Université de Montréal, Montréal QC, H2V 0B3 Canada

**Keywords:** Materials chemistry, Soft materials

## Abstract

For millennia, humans have exploited the special properties of porous materials. Advances in recent years have yielded a new generation of finely structured porous materials that allow processes to be controlled at the molecular level. These materials are built by a strategy of modular construction, using molecular components designed to position their neighbors in ways that create predictable voids.

## Abstract (web summary)

The strategy of modular construction makes it possible to create ordered materials with structures that are controlled at the molecular level. In this perspective article, the author shows how the approach has yielded a new generation of materials with extraordinary properties of porosity.

Nothing exists but atoms and the void.

Democritus

## The enduring utility of porous materials

For millenia, humans have relied on the uniquely useful properties of porous materials, such as the breathability of leather clothing, the special insulative value of furs with hollow hairs, and the high buoyancy of wood. These classic biomaterials have irregular structures and are considered to be macroporous, with openings wider than 500 Å. As technology has advanced, materials with more order, better-defined compositions, and networks of more finely structured pores have been discovered, both in nature and in the laboratory. During the last century, ordered materials of this type, with pore diameters in the ranges <20 Å (micropores) or 20–500 Å (mesopores), have become indispensable in modern life because their dimensions allow processes to be controlled at the molecular level.

## Zeolites as prototypic ordered microporous materials

**Fig. 1:**
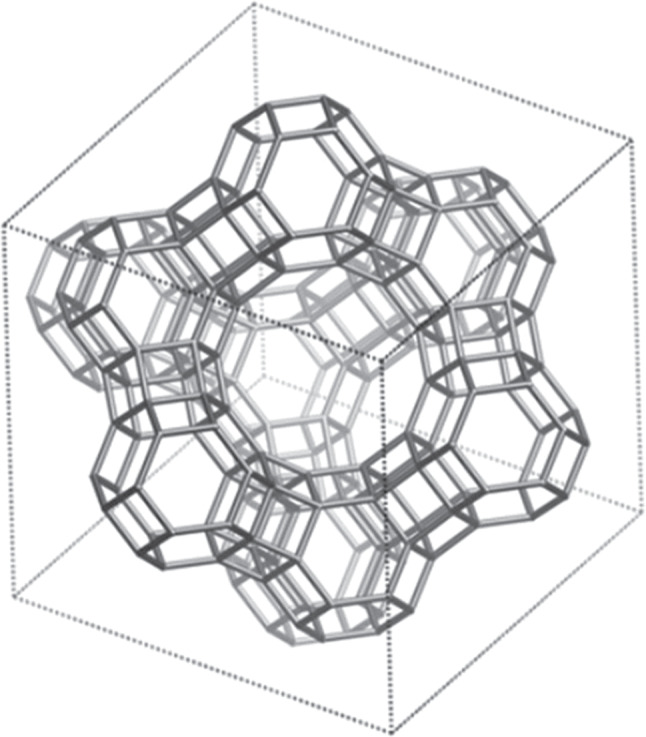
Representative structure of the faujasite series of zeolites. Nodes in the framework correspond to atoms of Al or Si, linked to shared atoms of O that are not shown explicitly. Ions and neutral guests in the cages and channels are omitted for simplicity. Adapted with permission from the Royal Society of Chemistry, ref. ^6^.

Naturally occurring crystalline aluminosilicate minerals called zeolites are prototypic ordered microporous solids. They were first reported in the scientific literature by Cronstedt in 1758 (ref. ^1^) and later found to sorb liquids and gases selectively and reversibly^2^. The first structural studies of single crystals of zeolites, published in 1930 by Taylor^3^ and Pauling^4,5^, established that nominally tetrahedral AlO_4_ and SiO_4_ units are linked by shared atoms of oxygen to form open structures, typically anionic frameworks surrounding exchangeable counterions and neutral guests. For example, Fig. 1 shows the open framework of the faujasite series of zeolites and reveals the characteristic presence of internal cages and interconnecting channels^6^.

Later pioneering studies by Barrer, Milton, Breck, and others explored the sorptive properties of zeolites and established new ways to make naturally occurring examples and unnatural analogs in the laboratory. Zeolites have become ubiquitous in modern technology, and their porosity makes them particularly useful in separation, ion exchange, sorption, and catalysis. Solids used for such applications do not necessarily have to be crystalline, and various amorphous porous materials offer attractive alternatives^7–9^. However, the well-ordered structures of zeolites confer unique advantages, including higher levels of selectivity arising from the uniformity of cages and channels.

Despite proven utility, zeolites have significant shortcomings. Many examples described in compilations such as the *Atlas of Zeolite Structure Types* are not robust enough to exist as empty frameworks when guests are removed, so they are not truly porous materials. Fragility can arise in part because the frameworks are held together by Si–O–Si bonding or related links, which can adopt a range of bond angles and yield rings and other assemblies with diverse sizes and shapes^10,11^. For these reasons, millions of potential zeolitic structures are energetically accessible, but only about 240 distinct, fully ordered forms with four-connected tetrahedral centers have been characterized so far, despite almost a century of synthesis and structural analysis^12^. No broadly effective strategy provides access to latent zeolitic diversity. Moreover, the percentage of volume accessible to guests within standard zeolites is modest (e.g., only about 60% in a typical member of the faujasite series), and the surface area in the same material is relatively small (about 1000 m^2^/g, as determined by Brunauer–Emmett–Teller (BET) analysis of the adsorption of N_2_ at 77 K)^13^. These shortcomings have prompted an active search for substitutes that are more porous, more structurally diverse, and more susceptible to rational synthesis.

## Modular construction of a new generation of porous ordered materials

During the last three decades, modular construction has been established as an effective way to make new materials with predetermined structures and properties, and it has proven to be particularly suitable for building porous solids. The strategy uses preformed molecular modules that are designed to be connectable in specific ways. The origin of the approach can be traced to two parallel advances in organic and inorganic chemistry^14^, one showing that predictably ordered networks can be built from modules that engage in directional noncovalent interactions such as hydrogen bonds^15–18^, and the other demonstrating that coordinative bonds to metals can also be used to control construction^19^. When modules are devised to be poorly flexible and to hold their neighbors in particular positions, the resulting materials cannot usually achieve effective packing and optimal intermolecular bonding at the same time. Open structures are thereby formed, with internal volumes occupied by molecules of solvent or other guests present during assembly.

These insights have triggered the explosive development of new post-zeolitic materials with unprecedented porosity, including metal-organic frameworks (MOFs)^20–22^, covalent organic frameworks (COFs)^23–28^, and hydrogen-bonded organic frameworks (HOFs)^29–32^. Much of this work has been carried out during the last decade. Although these new materials have been given different names, they are all constructed by processes that are conceptually identical: suitably designed molecular modules are prepared by chemical synthesis, and properly oriented divergent functional groups interact under the conditions of assembly to hold adjacent modules together in ways that create voids.

## In showing the utility of modular construction, MOFs help lead the way

**Fig. 2:**
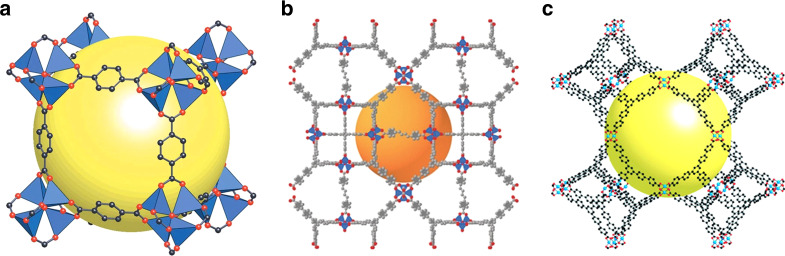
Structures of selected MOFs. **a** Structure of MOF-5, showing how ZnO_4_ tetrahedra (blue) are joined by 1,4-benzenedicarboxylate linkers (O in red and C in black) to give a cubic framework. The yellow surface represents the largest sphere (diameter 12 Å) that can occupy pores without contacting the van der Waals surface of the framework. Adapted with permission from Springer Nature, ref. ^20^. **b** Structure of DUT-60, with ZnO_4_ tetrahedra shown in blue and with atoms of the linkers in red (oxygen) and gray (carbon). The orange surface represents the largest sphere (diameter 36 Å) that can occupy the largest pores without contacting the van der Waals surface of the framework. Adapted with permission from John Wiley & Sons, Inc. (Wiley), ref. ^40^. **c** Structure of MOF-399, with atoms shown as in **a**. The yellow surface represents the largest sphere (diameter 43 Å) that can occupy the largest pores without contacting the van der Waals surface of the framework. Adapted with permission from the American Chemical Society (ACS), ref. ^43^. In all three figures, atoms of hydrogen have been omitted for clarity.

The remarkable properties of MOFs have attracted widespread interest in the field of porous materials. A key insight was to replace simple coordinative bonds to metals by more solid and bulkier connections involving multiple metals. In the construction of prototypic MOF-5 (Fig. 2a), for example, zinc carboxylate units of the type Zn_4_O(OOCR)_6_ are formed when 1,4-benzenedicarboxylic acid reacts with Zn(NO_3_)_2_ under defined conditions, yielding a robust cubic *Fm*-3*m* network with metallic nodes and organic linkers^33^. Guest molecules introduced during assembly can be removed by heating under vacuum to create a rigorously porous crystalline network with a surface area of 3600 m^2^/g, as determined by BET analyses of anhydrous samples^34,35^.

Following the initial report of MOF-5 in 1999, systematic alteration of nodes and linkers has yielded many thousands of new MOFs with widely varying topologies and striking increases in surface area^36^. In 2012, for example, NU-110 was reported to have a specific surface area nearly twice as large (7140 m^2^/g)^37^, created through (1) the clever deployment of acetylenic linkers, (2) a gentle new technique for removing guests using supercritical CO_2_, and (3) a network topology that precludes internal volume from being reduced by the possibility of interpenetration, in which an open framework encloses one or more independent frameworks of the same or different types^38,39^. Obviously, interpenetration decreases porosity; however, it also has advantages, such as the stabilization of open frameworks, fine adjustment of pore sizes, and the introduction of dynamic effects resulting when interpenetrated networks are repositioned.

The current MOF record holder, DUT-60 (Fig. 2b), has a BET surface area of 7840 m^2^/g^40^. The structural characterization of DUT-60 is based on powder X-ray diffraction (PXRD) rather than on the analysis of single crystals, as in the case of MOF-5, NU-110, and many other frameworks. The percentage of guest-accessible volume in DUT-60 (90%, as assessed by PLATON^41,42^) and the calculated density of guest-free material (0.187 g/cm^3^) are only slightly lower than those of MOF-399 (Fig. 2c), which has an accessible volume of 94% and a calculated density of 0.126 g/cm^3^ (ref. ^43^). Until the last decade, these remarkable figures of merit would have been considered by many researchers to be unattainable in porous ordered materials.

**Fig. 3:**
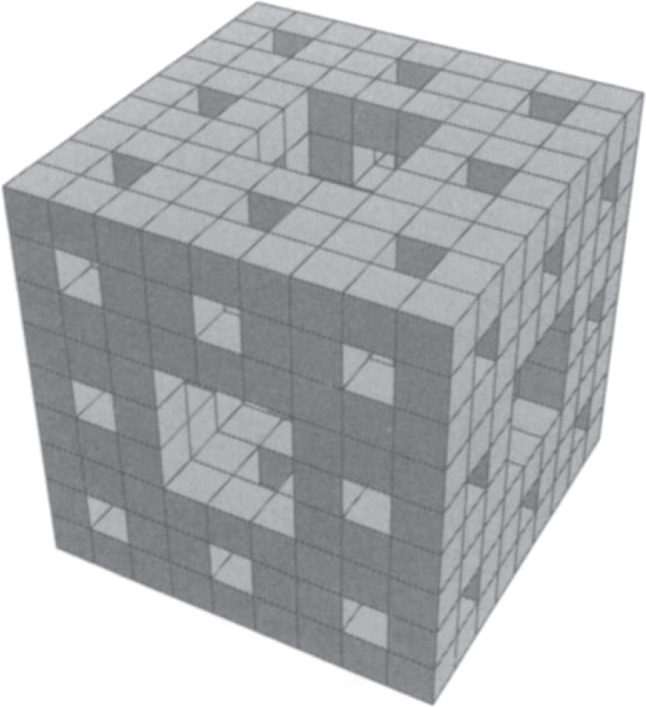
Construction of a Menger sponge. Adapted with permission from Taylor & Francis, Ltd., ref. ^45^.

MOF-399 was described by Furukawa et al. as porous, although no evidence has been presented to show that the framework is robust enough to exist in guest-free form. In fact, few MOFs with similarly low calculated densities are known to have the mechanical stability needed to allow guests to be removed to give empty solids^44^. The upper limits of porosity in crystalline solids remain unclear. It is instructive to compare the modular construction of increasingly porous solids with the process of creating a Menger sponge (Fig. 3)^45^, a mathematical object first described by Karl Menger in 1926^46^. Construction of a Menger sponge can be considered to start with a cube divided into 27 identical smaller cubes. The central small cube on each of the six faces is removed, along with the small cube at the center of the object. Each of the remaining small cubes is itself then divided into 27 even smaller cubes, the same seven central cubes are removed, and the process is continued to infinity. As this process proceeds, the volume of the object approaches zero, and its surface area increases without bound.

Of course, real solids cannot be subdivided indefinitely, so specific surface area has an upper limit. Similarly, increasingly porous MOFs cannot simply be made by elongating the constituent modules. For example, replacing 1,4-benzenedicarboxylate linkers in MOF-5 (Fig. 2a) with longer *p*-phenylenedicarboxylates is expected to lead to frameworks with a maximum BET surface area similar to that of poly(*p*-phenylene) itself, which is about 10,500 m^2^/g^47^. Modest improvements can be made by using acetylenic linkers in place of *p*-phenylenes, as illustrated by the properties of NU-110 (ref. ^37^). Nevertheless, a theoretical ceiling exists, and approaching it will require overcoming a host of obstacles, including the synthesis, purification, and dissolution of suitable elongated modules. Moreover, it is not obvious that the pursuit of ever higher specific surface areas will necessarily lead to materials of greater utility. However, valuable insights about how to make the most effective use of modular construction will continue to emerge.

## Modular construction of COFs

**Fig. 4:**
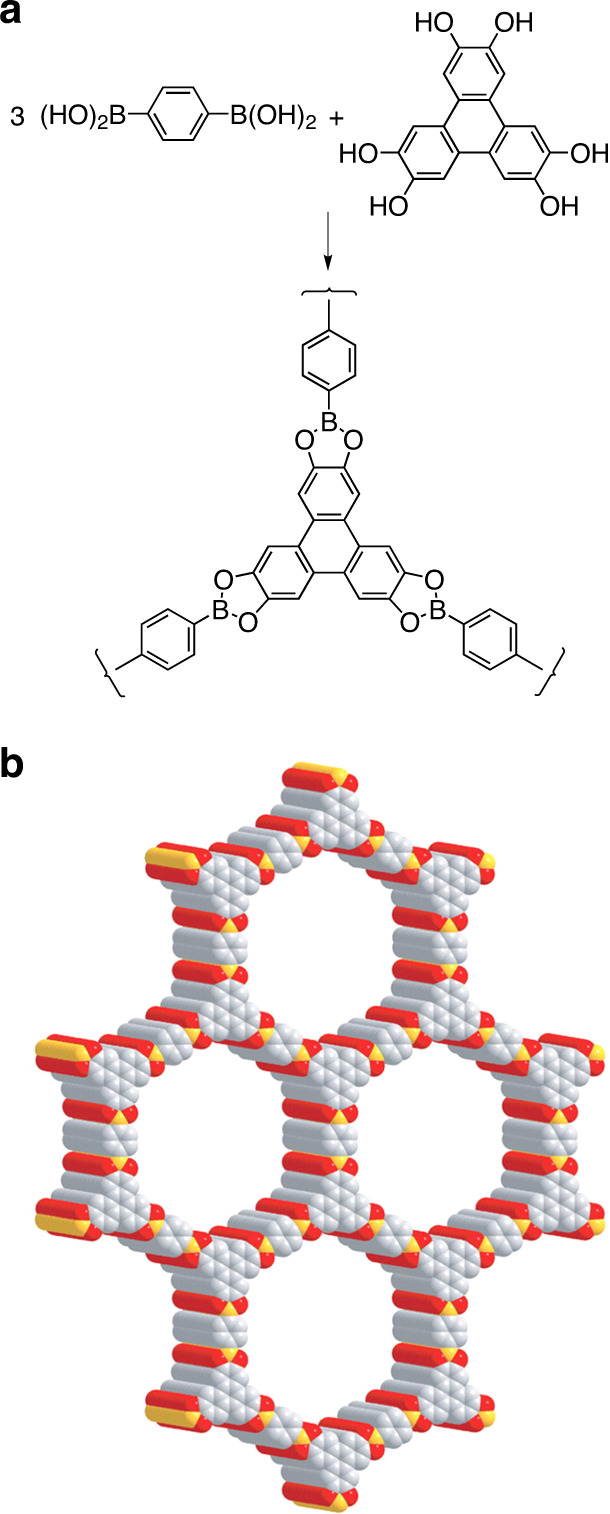
Structure of COF-5. **a** Condensation of 1,4-benzenediboronic acid with 2,3,6,7,10,11-hexahydroxytriphenylene to form COF-5. **b** Model of the graphitic structure of COF-5 based on PXRD, with atoms of carbon shown in gray, boron in yellow, and oxygen in red. Atoms of hydrogen are omitted for clarity. Adapted with permission from the American Association for the Advancement of Science (AAAS), ref. ^48^.

Like MOFs, COFs are porous crystalline framework structures, but the components are linked by covalent bonds involving light elements, rather than by coordinative interactions with metals. A prototypic example is COF-5 (Fig. 4a)^48^, a boronate formed along with water when 1,4-benzenediboronic acid condenses with 2,3,6,7,10,11-hexahydroxytriphenylene. Together, the linearity of the diboronic acid, the trigonal geometry of the triphenylene, and the directionality of intermodular bonding are designed to program the formation of open graphitic sheets. However, obtaining a well-ordered product requires striking a delicate balance between removing water to favor condensation, and retaining water to make condensation reversible and to ensure that errors in assembly are corrected. Under carefully defined conditions, condensation can be induced to form a microcrystalline solid, and analysis by PXRD suggests that the structure consists of the expected graphitic sheets, stacked in superposition to create a hexagonal array of parallel mesopores with a diameter of 27 Å (Fig. 4b). Guest molecules incorporated during assembly can be removed by heating under vacuum to create a porous structure with retained crystallinity and a BET surface area of 1590 m^2^/g.

**Fig. 5:**
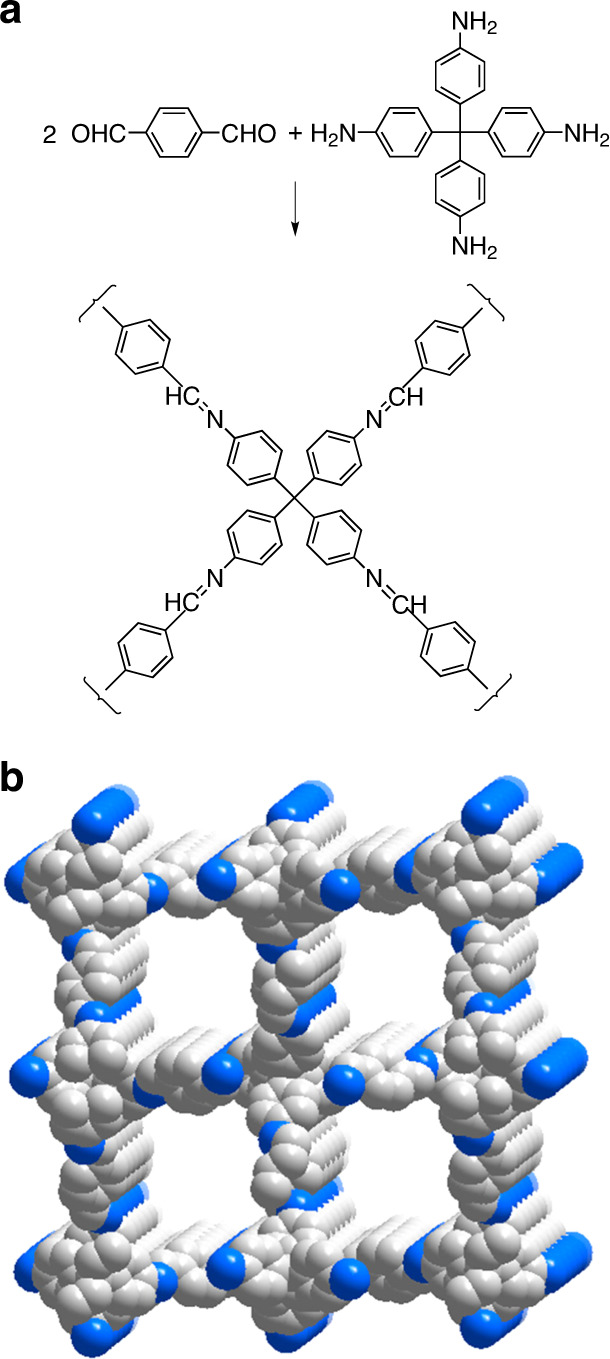
Structure of COF-300. **a** Condensation of 1,4-benzenedicarboxaldehyde with tetrakis(4-aminophenyl)methane to form COF-300. **b** Model of the postulated fivefold interpenetrated diamondoid structure of COF-300 based on PXRD, showing channels along the *c*-axis. Atoms of carbon are shown in gray, atoms of nitrogen appear in blue, and atoms of hydrogen are omitted for clarity. Adapted with permission from ACS, ref. ^28^.

In general, boronates, boroxines, and related compounds of boron are easily hydrolyzed, so subsequent development of the field of COFs has targeted more robust classes of compounds, most notably imines formed by condensing aryl-substituted aldehydes with amines^49^. A representative example is COF-300 (Fig. 5a)^50^, a poly(imine) formed from 1,4-benzenedicarboxaldehyde and tetrakis(4-aminophenyl)methane. Microcrystalline samples were obtained under defined conditions, as confirmed by PXRD, and an extensive process of modeling, refining, and comparing experimental and simulated diffraction patterns suggested that COF-300 has a fivefold interpenetrated diamondoid structure (Fig. 5b). Parallel channels with dimensions of 7.8 × 7.8 Å^2^ lie along the *c*-axis, and guests can be removed without loss of crystallinity, giving a porous framework with a BET surface area of 1360 m^2^/g and a measured density of 0.66 g/cm^3^.

In cases with simple topologies such as COF-5 (graphitic framework) and COF-300 (diamondoid framework), data from PXRD can often be analyzed to produce acceptable structural models. However, fuller development of the field is held back by the inherent difficulty of growing single crystals of covalently bonded materials, which is a prerequisite for obtaining structural data of the highest quality. In contrast, crystallization is often straightforward when modular construction is more readily reversible, as in the case of MOFs. Without single crystals, structural analyses of complex COFs are exceedingly challenging, geometric details are not readily accessible, and important phenomena such as interpenetration and disorder cannot be properly examined. As a result, major effort has been devoted to finding ways to produce single crystals of open covalently bonded networks.

**Fig. 6:**
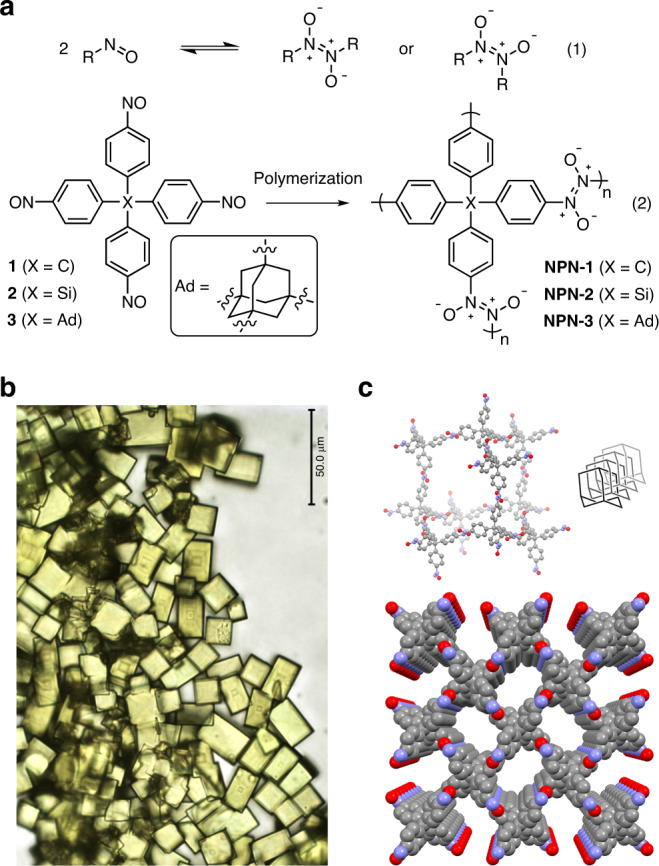
Producing single crystals of a covalently bonded network. **a** Conversion of nitroso compounds into azodioxy dimers and polymers. **b** Crystals of the azodioxy polymer derived from nitroso monomer **1**. Adapted with permission from Springer Nature, ref. ^52^. **c** Fourfold interpenetrated diamondoid structure of the polymer, as determined by single-crystal XRD. Channels appear along the *c*-axis, guests and atoms of hydrogen are omitted for clarity, and other atoms are shown in standard colors. Adapted with permission from Springer Nature, ref. ^52^.

This elusive goal was first reached by using the reaction of nitroso compounds to give azodioxy dimers, which is a reversible process (Eq. 1)^51^. Tetrahedral tetranitroso monomers **1**–**3** react in solution to produce the expected azodioxy polymers (Eq. 2) as large single crystals (Fig. 6a), which were shown by XRD to consist of open interpenetrated diamondoid networks (Fig. 6b)^52^. In the polymer derived from monomer **1**, for example, the guest-accessible volume is 36%, and channels with dimensions of 7.3 × 3.3 Å^2^ lie along the *c*-axis. Guests can be removed by heating under vacuum, but the azodioxy links do not appear to be robust enough to allow the formation of guest-free frameworks with retention of crystallinity.

**Fig. 7:**
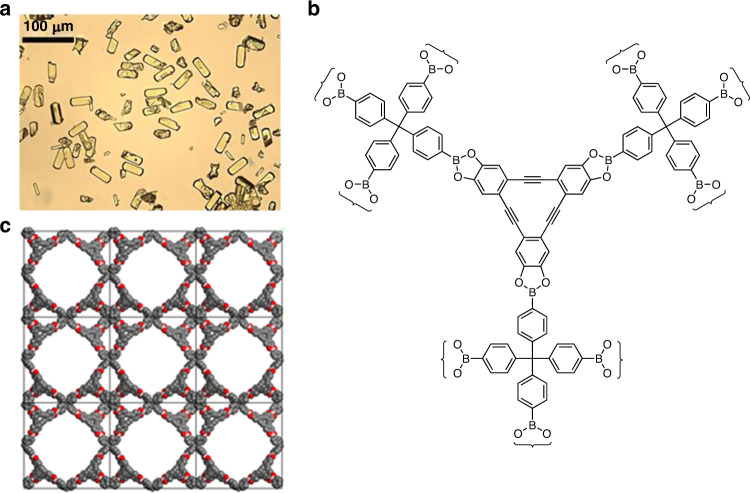
Images of selected COFs. **a** Micrograph showing single crystals of COF-300. Adapted with permission from AAAS, ref. ^54^. **b** Structure of DBA-3D-COF 1. **c** Open network of DBA-3D-COF 1, as determined by PXRD. Adapted with permission from ACS, ref. ^55^.

The strategy of modular construction makes it possible to devise new COFs with foreseeable structural features, but details of assembly, including how initial crystallites are formed and grow, remain obscure and may vary according to the type of covalent bonds formed^53^. Better understanding of these phenomena is likely to reveal how structural order can be enhanced in rational ways, such as by adding agents that inhibit nucleation and modulate crystallization. A remarkable example is the preparation of COF-300 (Fig. 5) in the presence of excess aniline, which competes with tetrakis(4-aminophenyl)methane for condensation with 1,4-benzenedicarboxaldehyde^54^. Under these special conditions, large single crystals of COF-300 could be grown during 1–2 months (Fig. 7a), and structural analysis by XRD revealed that the diamondoid network is sevenfold interpenetrated, whereas previous models of COF-300 based on PXRD had suggested fivefold interpenetration^50^. It is possible that the two methods of preparation give rise to materials with different degrees of interpenetration. However, the discrepancy underscores the importance of learning how to produce MOFs and COFs in the form of single crystals suitable for detailed structural analysis.

Covalent bonding and the exclusive presence of light elements make COFs inherently robust, lightweight, and more suitable than MOFs for applications requiring porous ordered materials with high thermal stability and low density. These advantages have motivated an active search for new COFs, and materials with remarkable properties continue to emerge. A noteworthy recent example is DBA-3D-COF 1 (Fig. 7b, c), a boronate that forms an open network with the lowest density (0.13 g/cm^3^) and the highest BET surface area (5083 m^2^/g) reported so far for a COF^55^.

## HOFs take their place alongside MOFs and COFs

During the last decade, the flood of fascinating new MOFs and COFs has been swollen by a torrent of HOFs and related porous materials built from modules connected by hydrogen bonds or other directional noncovalent interactions. HOFs are important because their properties are complementary to those of MOFs and COFs. In particular, the modular components of HOFs are lightweight, metal-free, and easily crystallized. They can be dissolved, purified rigorously by standard methods, and processed by low-energy solution-based techniques. In addition, it may be possible to make biodegradable and biocompatible HOFs from renewable resources such as biomass. Because hydrogen bonds are weaker than coordinative interactions and covalent bonds, HOFs can be expected to be more deformable than MOFs and COFs and to show unique selectivity in accommodating guests. Simultaneously, however, the relative weakness of hydrogen bonds makes the modular construction of HOFs with significant levels of permanent porosity inherently challenging.

Because molecules have irregular shapes and convoluted surfaces, packing in crystals is inherently inefficient, and only about 70% of the volume is occupied by the constituent atoms. Normally, the voids are widely disseminated in the form of cavities too small to accommodate guests. An instructive exception to this rule is the behavior of hydroquinone^56^, which crystallizes in the β-form as an open hydrogen-bonded network in which about 16% of the volume is accessible to small guests, such as solvents. Crystals can be obtained either in porous guest-free form or as solvates. Although the porosity is low, it arises from modules of great simplicity, underscoring the potential of more complex HOFs to serve as porous materials.

**Fig. 8:**
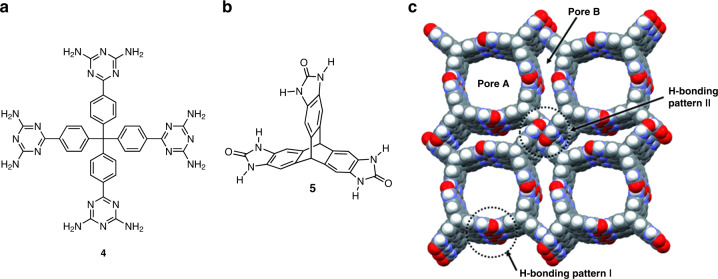
Structures of selected HOFs. **a** Molecular structure of tetrakis(diaminotriazine) **4**. **b** Molecular structure of triptycenetrisimidazolone **5**. **c** Structure of a HOF derived from compound **5**. Adapted with permission from Wiley, ref. ^61^.

A breakthrough in the field was the demonstration that crystalline hydrogen-bonded molecular solids can have high porosities like those of zeolites. In this work, crystals of tetrakis(diaminotriazine) **4** (Fig. 8a) were desolvated under vacuum to give a porous solid without loss of crystallinity, as confirmed initially by single-crystal XRD and later by porosimetry^57,58^. The BET surface area is modest (359 m^2^/g), but sorption proved to be highly selective, as shown by the ability to surpass MOFs in distinguishing between similar pairs such as C_2_H_2_/C_2_H_4_. This highlights the special ability of HOFs to alter their structures in ways that conform to potential guests^59,60^.

Subsequent work has yielded many new HOFs with exceptional properties of porosity. The state of the art was notably advanced by the discovery that triptycenetrisimidazolone **5** (Fig. 8b) forms a HOF with a BET surface area of 2796 m^2^/g and an ability to sorb CO_2_ (15.9 wt% at 273 K/1 bar) and H_2_ (2.2 wt% at 77 K/1 bar) similar to that of MOFs (Fig. 6c)^61–63^. A more open crystalline form of compound **5** has been reported to have a BET surface area of 3425 m^2^/g and a density of only 0.413 g/cm^3^, which is the lowest value reported so far in the Cambridge Structural Database for any molecular solid^64^. As a result, HOFs are no longer mere curiosities, and they stand alongside MOFs and COFs as the most remarkable porous ordered materials known.

## MOFs, COFs, and HOFs are joined by other exciting new porous ordered materials

Emphasizing framework structures such as MOFs, COFs, and HOFs in an account of recent advances in the field of porosity does not imply that other ordered porous materials are less promising. In typical MOFs, COFs, and HOFs, porosity arises chiefly from the nature of modular construction, and the individual modules are not intrinsically porous. An alternative is to build ordered materials that are porous primarily because they are composed of molecular cages that enclose volumes large enough to accommodate smaller guests, or because packing is unusually inefficient^65–70^. During the last decade, relatively simple ways to make molecular cages and compounds with particularly awkward shapes have been devised, and crystalline solids derived from them have shown remarkable levels of porosity, approaching in a few cases those of MOFs, COFs, and HOFs^69^.

## Summary

The development of materials with robust open frameworks has accelerated dramatically in the last decade, and it is clear that MOFs, COFs, HOFs, and their analogs will play key roles in science and technology for many years to come. Their unique properties, including unprecedented levels of porosity, set them well apart from all materials available in the past. Methods for large-scale production have been devised, various MOFs are now available in bulk from suppliers, and commercial applications are emerging, particularly in the capture, storage, and release of gases and other small molecules. Directly underlying the development of these new materials is the concept of modular construction, which plays a critical role by guiding syntheses toward the formation of well-ordered open frameworks, by permitting desired structures to be made by design, and by allowing properties to be tailored rationally according to need. The modules and the interactions that hold them together can be varied extensively, making the scope of modular construction virtually unlimited.

The rapidly growing field will expand even faster as tools for the structural characterization of microcrystalline solids are improved, such as through advances in PXRD or electron diffraction^71^, and as new ways are devised to obtain the products of modular construction in the form of easily characterized single crystals. Better ways are also needed to reinforce open frameworks produced by modular construction and to inhibit loss of crystallinity when guests are exchanged or removed^72^. In addition, detailed understanding of the mechanisms of construction is likely to be a prolific source of fresh ideas^53^. It is also important to continue to broaden the scope of modular construction to include ordered solids that are not merely porous, but have the widest possible range of functionality^73–76^. Examining the structures of MOFs, COFs, and HOFs that have noteworthy properties, such as the examples in Figs. 2 and 4–8, reveals that the carbon-based modules from which they are built are often complex molecules synthesized by multistep routes. An urgent goal is to learn how to make other exceptional ordered materials by simpler methods of synthesis, ideally using only inexpensive modular components that are derived from renewable resources by green methods^77–82^. Together, these challenges and opportunities ensure that modular construction will continue to be a highly dynamic area of science and technology.

## Data Availability

No datasets were generated or analyzed during the current study.

## References

[1] Cronstedt AF (1756). Ron och beskriting om en obekant bärgant, som kallas zeolites. Akad. Handl. Stockh..

[2] Flanigen, E. M., Broach, R. W. & Wilson, S. T. in *Zeolites in Industrial Separation and Catalysis* 1−26 (Wiley-VCH, Weinheim, 2010).

[3] Taylor WH (1930). The structure of analcite (NaAlSi_2_O_6_ H_2_O). Z. Kristallogr. Cryst. Mater..

[4] Pauling L (1930). The structure of some sodium and calcium aluminosilicates. Proc. Natl Acad. Sci. USA.

[5] Pauling L (1930). The structure of sodalite and helvite. Z. Kristallogr. Cryst. Mater..

[6] Verboekend D (2016). Synthesis, characterisation, and catalytic evaluation of hierarchical faujasite zeolites: milestones, challenges, and future directions. Chem. Soc. Rev..

[7] Bennett TD, Horike S (2018). Liquid, glass and amorphous solid states of coordination polymers and metal-organic frameworks. Nat. Rev. Mater..

[8] Kupgan G, Abbott LJ, Hart KE, Colina CM (2018). Modeling amorphous microporous polymers for CO_2_ capture and separations. Chem. Rev..

[9] Tian W (2020). Porous carbons: structure-oriented design and versatile applications. Adv. Funct. Mater..

[10] Baur WH (1992). Self-limiting distortion by antirotating hinges is the principle of flexible but noncollapsible frameworks. J. Solid State Chem..

[11] Wragg DS, Morris RE, Burton AW (2008). Pure silica zeolite-type frameworks: a structural analysis. Chem. Mater..

[12] The Zeolite Framework Database. http://www.iza-structure.org/databases/ (2017).

[13] Bae Y-S, Yazaydin AÖ, Snurr RQ (2010). Evaluation of the BET method for determining surface areas of MOFs and zeolites that contain ultra-micropores. Langmuir.

[14] Rosseinsky MJ (2002). Recent developments in metal-organic framework chemistry: design, discovery, permanent porosity and flexibility. Micropor. Mesopor. Mater..

[15] Ermer O (1988). Fivefold-diamondoid structure of adamantane-1,3,5,7-tetracarboxylic acid. J. Am. Chem. Soc..

[16] Ducharme Y, Wuest JD (1988). Use of hydrogen bonds to control molecular aggregation. Extensive, self-complementary arrays of donors and acceptors. J. Org. Chem..

[17] Simard M, Su D, Wuest JD (1991). Use of hydrogen bonds to control molecular aggregation. Self-assembly of three-dimensional networks with large chambers. J. Am. Chem. Soc..

[18] Etter MC (1990). Encoding and decoding hydrogen-bond patterns of organic compounds. Acc. Chem. Res..

[19] Hoskins BF, Robson R (1989). Infinite polymeric frameworks consisting of three dimensionally linked rod-like segments. J. Am. Chem. Soc..

[20] Yaghi OM (2003). Reticular synthesis and the design of new materials. Nature.

[21] Furukawa H, Cordova KE, O’Keeffe M, Yaghi OM (2013). The chemistry and applications of metal-organic frameworks. Science.

[22] Griffin SL, Champness NR (2020). A periodic table of metal-organic frameworks. Coord. Chem. Rev..

[23] Geng, K. et al. Covalent organic frameworks: design, synthesis, and functions. *Chem. Rev*. **120**, 10.1021/acs.chemrev.9b00550 (2020). 10.1021/acs.chemrev.9b00550 31967791

[24] Guan X, Chen F, Fang Q, Qiu S (2020). Design and applications of three dimensional covalent organic frameworks. Chem. Soc. Rev..

[25] Lohse MS, Bein T (2018). Covalent organic frameworks: structures, synthesis, and applications. Adv. Funct. Mater..

[26] Diercks CS, Yaghi OM (2017). The atom, the molecule, and the covalent organic framework. Science.

[27] Bisbey RP, Dichtel WR (2017). Covalent organic frameworks as a platform for multidimensional polymerization. ACS Cent. Sci..

[28] Waller PJ, Gándara F, Yaghi OM (2015). Chemistry of covalent organic frameworks. Acc. Chem. Res..

[29] Yang J (2020). Porous hydrogen-bonded organic frameworks (HOFs): from design to potential applications. Chem. Eng. J..

[30] Hisaki I, Xin C, Takahashi K, Nakamura T (2019). Designing hydrogen-bonded organic frameworks (HOFs) with permanent porosity. Angew. Chem. Int. Ed..

[31] Lin R-B (2019). Multifunctional porous hydrogen-bonded organic framework materials. Chem. Soc. Rev..

[32] Luo J, Wang J-W, Zhang J-H, Lai S, Zhong D-C (2018). Hydrogen-bonded organic frameworks: design, structures and potential applications. CrystEngComm.

[33] Eddaoudi M (2002). Systematic design of pore size and functionality in isoreticular MOFs and their application in methane storage. Science.

[34] Walton KS, Snurr RQ (2007). Applicability of the BET method for determining surface areas of microporous metal−organic frameworks. J. Am. Chem. Soc..

[35] Kaye SS, Dailly A, Yaghi OM, Long JR (2007). Impact of preparation and handling on the hydrogen storage properties of Zn_4_O(1,4-benzenedicarboxylate)_3_ (MOF-5). J. Am. Chem. Soc..

[36] Moghadam PZ (2017). Development of a Cambridge Structural Database subset: a collection of metal-organic frameworks for past, present, and future. Chem. Mater..

[37] Farha OK (2012). Metal-organic framework materials with ultrahigh surface areas: Is the sky the limit?. J. Am. Chem. Soc..

[38] Baburin IA, Blatov VA, Carlucci L, Ciani G, Proserpio DM (2008). Interpenetrated three-dimensional networks of hydrogen-bonded organic species: a systematic analysis of the Cambridge Structural Database. Cryst. Growth Des..

[39] Jiang H-L, Makal TA, Zhou H-C (2013). Interpenetration control in metal–organic frameworks for functional applications. Coord. Chem. Rev..

[40] Hönicke IM (2018). Balancing mechanical stability and ultrahigh porosity in crystalline framework materials. Angew. Chem. Int. Ed..

[41] Spek, A. L. *PLATON, A Multipurpose Crystallographic Tool* (Utrecht University, Utrecht, The Netherlands, 2001).

[42] van der Sluis P, Spek AL (1990). BYPASS: an effective method for the refinement of crystal structures containing disordered solvent regions. Acta Crystallogr..

[43] Furukawa H (2011). Isoreticular expansion of metal–organic frameworks with triangular and square building units and the lowest calculated density for porous crystals. Inorg. Chem..

[44] Barbour, L. J. Crystal porosity and the burden of proof. *Chem. Commun*. 1163–1168 (2006). 10.1039/b515612m 16518481

[45] Zhou L, Mackeprang C, Myers K (2007). Coloring graphs on sponges. Am. Math. Monthly.

[46] Menger, K. in *Classics on Fractals (Studies in Nonlinearity)* (ed. Edgar, G. A.) (Westview Press, Advanced Book Program, Boulder, CO, 2004).

[47] Schnobrich JK, Koh K, Sura KN, Matzger AJ (2010). A framework for predicting surface areas in microporous coordination polymers. Langmuir.

[48] Côté AP (2005). Porous, crystalline, covalent organic frameworks. Science.

[49] Segura JL, Mancheño MJ, Zamora F (2016). Covalent organic frameworks based on Schiff-base chemistry: synthesis, properties and potential applications. Chem. Soc. Rev..

[50] Uribe-Romo FJ (2009). A crystalline imine-linked 3-D porous covalent organic framework. J. Am. Chem. Soc..

[51] Beaudoin D, Wuest JD (2016). Dimerization of aromatic *C*‑nitroso compounds. Chem. Rev..

[52] Beaudoin D, Maris T, Wuest JD (2013). Constructing monocrystalline covalent organic networks by polymerization. Nat. Chem..

[53] Li H (2020). Nucleation–elongation dynamics of two-dimensional covalent organic frameworks. J. Am. Chem. Soc..

[54] Ma T (2018). Single-crystal X-ray diffraction structures of covalent organic frameworks. Science.

[55] Baldwin LA, Crowe JW, Pyles DA, McGrier PL (2016). Metalation of a mesoporous three-dimensional covalent organic framework. J. Am. Chem. Soc..

[56] Palin, D. E. & Powell, H. M. The structure of molecular compounds. Part VI. The β-type clathrate compounds of quinol. *J. Chem. Soc*. 815–821 (1948). 10.1039/jr9480000571 18869429

[57] Brunet P, Simard M, Wuest JD (1997). Molecular tectonics. Porous hydrogen-bonded networks with unprecedented structural integrity. J. Am. Chem. Soc..

[58] He Y, Xiang S, Chen B (2011). A microporous hydrogen-bonded organic framework for highly selective C_2_H_2_/C_2_H_4_ separation at ambient temperature. J. Am. Chem. Soc..

[59] Cui P (2020). An expandable hydrogen-bonded organic framework characterized by three-dimensional electron diffraction. J. Am. Chem. Soc..

[60] Lü J (2014). A robust binary supramolecular organic framework (SOF) with high CO_2_ adsorption and selectivity. J. Am. Chem. Soc..

[61] Mastalerz M, Oppel IM (2012). Rational construction of an extrinsic porous molecular crystal with an extraordinary high specific surface area. Angew. Chem. Int. Ed..

[62] Cooper AI (2012). Molecular organic crystals: from barely porous to really porous. Angew. Chem. Int. Ed..

[63] Li P (2019). Interpenetration isomerism in triptycene-based hydrogen-bonded organic frameworks. Angew. Chem. Int. Ed..

[64] Pulido A (2017). Functional materials discovery using energy–structure–function maps. Nature.

[65] Little MA, Cooper AI (2020). The chemistry of porous organic molecular materials. Adv. Funct. Mater..

[66] Beuerle F, Gole B (2018). Covalent organic frameworks and cage compounds: design and applications of polymeric and discrete organic scaffolds. Angew. Chem. Int. Ed..

[67] Mastalerz M (2018). Porous shape-persistent organic cage compounds of different size, geometry, and function. Acc. Chem. Res..

[68] Dalgarno SJ, Thallapally PK, Barbour LJ, Atwood JL (2007). Engineering void space in van der Waals crystals: calixarenes lead the way. Chem. Soc. Rev..

[69] McKeown NB (2020). Organic molecules of intrinsic microporosity. Org. Mater..

[70] Das S, Heasman P, Ben T, Qiu S (2017). Porous organic materials: strategic design and structure–function correlation. Chem. Rev..

[71] Gemmi M (2019). Electron diffraction: the nanocrystallography revolution. ACS Cent. Sci..

[72] Feriante CH (2020). Rapid synthesis of high surface area imine-linked 2D covalent organic frameworks by avoiding pore collapse during isolation. Adv. Mater..

[73] Rao MR, Fang Y, De Feyter S, Perepichka DF (2017). Conjugated covalent organic frameworks via Michael addition−elimination. J. Am. Chem. Soc..

[74] Ascherl L (2018). Solvatochromic covalent organic frameworks. Nat. Commun..

[75] Haug WK, Moscarello EM, Wolfson ER, McGrier PL (2020). The luminescent and photophysical properties of covalent organic frameworks. Chem. Soc. Rev..

[76] Wang B (2020). Microporous hydrogen-bonded organic framework for highly efficient turn-up fluorescent sensing of aniline. J. Am. Chem. Soc..

[77] Kandambeth S, Dey K, Banerjee R (2019). Covalent organic frameworks: chemistry beyond the structure. J. Am. Chem. Soc..

[78] Li Y, Chen W, Xing G, Jiang D, Chen L (2020). New synthetic strategies toward covalent organic frameworks. Chem. Soc. Rev..

[79] Reinsch, H. “Green” synthesis of metal-organic frameworks. *Eur. J. Inorg. Chem*. **2016**, 4290–4299 (2016).

[80] Karak S (2017). Constructing ultraporous covalent organic frameworks in seconds via an organic terracotta process. J. Am. Chem. Soc..

[81] Ajoyan Z, Marino P, Howarth AJ (2018). Green applications of metal–organic frameworks. CrystEngComm.

[82] Peh SB, Wang Y, Zhao D (2019). Scalable and sustainable synthesis of advanced porous materials. ACS Sustain. Chem. Eng..

